# Notes and News

**Published:** 1890-08-30

**Authors:** 


### Notes and News.

Collected and Communicated.

The annual report of St. Helen's Cottage Hospital is a good one. A horse ambulance is to be started for the use of patients, and it is also proposed to enlarge the building. The number of patients admitted during the year 1889 was 200, against 225 in 1888, and 176 in 1887. The committee thank the subscribers, clergy, and collectors at the works for their continued assistance and support.

Medical men have been figuring in the public press in a distressing manner lately: poor Dr. Townsend and the late Mr. De La Motte in a tragedy which is more or less involved in mystery. But once a scare is roused it is hard to reproduce peace, and Dr. Townsend had hardly got abroad in search of forgetfulness for his woes, when he was called back to attend an inquest on a friend who had died previously to the great tragedy. A witness in this case was Mr. Hart, surgeon at the docks. The morbid excitement caused in the districts where these sensational cases take place is something extraordinary, and the wonder is that more wild accusations are not made by the spread of gossip.

The Central London Throat and Ear Hospital issues a simple but excellent report. The in-patients admitted last year numbered 218, and 6,450 actually new out-patients have been treated. The hospital is in the Gray's Inn Road and has been in existence for 16 years: its long list of vice-presidents is guarantee of its continued efficiency; its balance-sheet is proof of its economy. Wines, spirits, and beer are down at £2 9s. Hospitals are terrible Oliver Twists, so here are the wants of the Central London Throat and Ear Hospital: (1) to erect a new out-patient department, entirely separate from that of the in-patients; (2) to make a recreation ground or garden for the in-patients, so as to avoid their taking exercise in the streets; (3) to so arrange and alter the present building as to afford improved ward accommodation, and to extend the nursing and domestic arrangements. We would suggest that when these improvements are carried out there should be a grand ceremony and speeches; the address of the Bishop of Durham in 1875 is a somewhat old story to tell in a report dated 1890.

Three new ambulance stations have been equipped by the Hospitals Association: the first on the cab rank in St. James's Square; the second on the cab rank in Pickering Place, Bayswater; and the third on the cab rank in Maida Vale. This brings the number of ambulance stations established by the Hospitals Association during the present year to fifty-one, which number will shortly be largely increased.

Noble's Isle of Man Hospital treated 259 in-patients last year, and 3,193 out-patients. The income was £1,786, which would hardly have covered expenses, but some exceptional donations were received. The island has just kept Hospital Sunday, and sermons warmly in favour of the hospital were preached. Notably was this the case at St. Mary's Roman Catholic Chapel, Father Walsh pointing out that during Miss Ransford's illness, a Catholic matron was appointed to fulfil her duties.

The tenth annual meeting of the British Dental Association took place at Exeter. The council suggested that the next year's meeting should be held at Bath, and Mr. E. L. Dudley be president-elect. A splendidly engraved salad bowl, containing a sum of money and accompanied by an illuminated address, was presented to Mr. Brownemason for his services rendered as secretary for the past seven years. There were some excellent papers read, and the Mayor and inhabitants of the town were not backward in social entertainments.

Bournemouth kept Hospital Saturday and Sunday on August 16th and 17th in fine weather, and the procession was a large one, and the collection in excess of last year. Service was held in St. Peter's Church, and Canon Fisher preached an appropriate sermon. The United Friendly Societies taking part were the Ancient Order of Foresters, the Oddfellows, the Hearts of Oak, and the Amalgamated Society of Carpenters and Joiners. This year the Hearts of Oak Society took the management, Mr. A. Parsons acting as hon. secretary.

The Royal Cornwall Infirmary has a deficit, and a growing number of out-patients; putting these two facts together, the governors have decided to make inquiries into the income, &c., of those who apply as out-patients. We recommend the secretary to study the plan in vogue at the London Hospital, which Mr. Nixon explained before the Lords' Committee. The infirmary treated 336 in-patients last year, and 900 out-patients. The average number of in-patients is 39, being two more than last year; and the cost per bed has been £48 16s. 4d., as compared with £48 9s. 9d., showing an increase of 6s. 8d. per bed. Sir Vyell Vyvyan was elected president.

An alleged case of Asiatic cholera at Poplar Hospital caused great excitement last week. The circumstances of the case have been investigated by Mr. W. H. Power, of the Medical Department of the Local Government Board. The patient, who had served as a coal trimmer on board the steamship "Duke of Argyll" which reached the Thames on Sunday the 17th inst., has been suffering from symptoms which, although clinically undistinguishable from true cholera, are from time to time observed in cases of cholera nostras such as occur annually at this season of the year in London and other large cities. The "Duke of Argyll" left Calcutta on June 21st, and Madras on June 30th, and did not touch at any port on her way home after she left Port Said on July 30th. Throughout the entire voyage from Calcutta up to the time when her crew left her on the 17th inst., no illness in any way resembling cholera had occurred on board. The patient, who was not attacked until upwards of twenty-four hours after leaving the vessel, is now on the mend, and will shortly be discharged.

The following vacancies are announced this week, the amount of the salary in each case being given after the name of the institution:—

**Resident Medical Officers.**

Taunton and Somerset Hospital, House Surgeon—£100, board, etc.

London Temperance Hospital, Junior House Surgeon—Board, etc.

Ramsgate Seaman's Infirmary, Medical Officer—£120, rooms, etc.

Birmingham General Hospital, Surgical Officer—£130, board, etc.

Suffolk General Hospital, House Surgeon—£80, board, etc.

Ryde Infirmary, Secretary and House Surgeon—£60, board, etc.

Wigan Albert Edward Infirmary, Junior House Surgeon—£80, board, etc.

Cheltenham Hospital Dispensary, Resident Surgeon—£180, rooms, etc.

Walsall Friendly Societies, Medical Officer—£200, rooms, etc.

**Non-Resident Medical Officers.**

Bristol Foresters' Medical Institute, Assistant Medical Officer—£150.

Infirmary for Consumption, Margaret Street, Physician-in-Ordinary.

---

After sitting for over a week the enquiry into the latest Hope Hospital scandal is concluded. The evidence was very unpleasant; the whole subject is one with which we do not care to deal, because it reflects shame on all such institutions more or less, and on the workers within them. Those who had to appear and give evidence have our sincere sympathy. The closing address of the inspector was as follows:—This inquiry was of great importance to the young men and young women whose characters had been assailed, but far beyond that it was of interest to that great institution. After the four or five inquiries which had been instituted there, more or less on the same subject, it ought to be not only the best institution in the locality, but all over England. The control of the hospital seemed principally to lie with the officials. He did not think any committee or board of guardians could keep things in order unless they had thorough confidence in their officers. He had now to present a careful and honest report of the inquiry to the Local Government Board, who would in due course communicate their decisions to the guardians."

---

One of the old Roman Emperors when informed that a man, dead of starvation, had been found within his dominions, fasted for a week as penance for permitting such a disgrace to be possible in his kingdom. Nowadays there is small concern about a death from starvation; a parliamentary report comes out every year telling its shameful tale—one just issued tells of 27 deaths in London from starvation in twelve months. Once a fortnight in the middle of this crowded metropolis of ours—surrounded by fellow-creatures who have enough and to spare—close to the bakers' shops, where the staff of life is piled up in such profusion—a human being sinks down and dies from sheer want of a crust of bread. These do not include the hundreds of deaths which are more or less consequent on poor food, bad housing, exposure, &c.; no; these are deaths simply from starvation. One woman, of 37, deserted by her husband, was convicted for sleeping in a cellar of an empty house without any visible means of subsistence, and was remanded. That night she appeared before another judge—she died of starvation. Indiscriminate charity won't remedy this evil; the sad, the bitter part of the story is that we cry in vain, that with the best will we scarcely can cope with this great giant of poverty. No law, no institution, can work so perfectly as to make starvation an absolute impossibility; only the ever widening sense of brotherhood, the hand of help and fellowship from man to man, can permanently reduce this evil. More or less we are all our brothers' keepers, but our English *gaucherie* and pride is too ready to make us pass by on the other side if we are rich, or go and die in a corner if we are poor, rather than acknowledge our common humanity and dependence on one another.

Cholera and scarlet fever are the chief topics of polite conversation at present, so here are "first remedies" for those persons to carefully read, mark, and learn who are anxious on the subject: "Cholera is generally preceded by symptoms which to get rid of very often suffices to prevent any further development of the disease. The most important of these symptoms is diarrhœa. As soon as diarrhœa shows itself a doctor should be called in, and while waiting for the doctor the patient should be put to bed and given nothing except hot tea, with rum or brandy added. An injection of a solution of starch, with, for adult persons, ten drops of laudanum of Sydenham added, is also efficacious."

---

A hospital sister puts in a plea for knitted socks as Christmas gifts for the adult wards. Her first request was for boots, but when we pointed out that our readers were not all shoemakers she replied, "Well then, ask them to knit me some nice warm socks for my men. It is so hard in the winter, when they are mostly ill with pneumonia, bronchitis, pleurisy, or cold in some form or another, to send them out without proper warm clothing." So will some of those readers who are kindly working for us turn their attention to men's socks. If any parcels are ready, and the workers want them out of the way, will they please send them to "Christmas," The Hospital Office, 140, Strand, London, W.C.

---

How is it that quack medicines always receive the support of the clergy? No new remedy for consumption, debility, or cancer is ever advertised without the names and addresses ot several ministers of religion who have been cured by this new medicine. Gradually one comes to believe that the clergy must all have been sufferers from diseases which qualified practitioners unkindly dub "incurable," but for which a remedy can be found by those who are not versed in pharmacy or therapeutics. A scornful physician of an Infirmary refuses to enlarge the wards and practice electro-homœopathy there, though he is called upon to do so in the public Press by a Baptist minister. In fact, this physician is rude enough to say that electro-homœopathy combines the elements of trade monopoly and charlatinism, both of which are incompatible with true humanity or its synonym Christianity. It is impossible to expect doctors to believe in a remedy which is "secret"—their scientific minds rebel, and it is their creed that any remedy God may provide for human ills is the property of all humanity, and is not to be used as a "trade monopoly." One would have thought that the clergy would have recognized a bond of fellowship here, but faith seems to run riot sometimes, and hence the number of reverend gentlemen who are ready to applaud the latest nostrum. Be it recorded, however, that two or three ministers in large towns, astonished at the prices their poor parishioners paid for sham electric belts, &c., have studied the subject, traced the belts to the makers, taken professional advice, and then waged war against the terrible waste of money.

